# Robotic liver resection improves self-perception of recovery compared to open approach in a quality-of-life survey

**DOI:** 10.1007/s13304-026-02717-4

**Published:** 2026-06-26

**Authors:** Paolo Magistri, Beatrice Pelloni, Giuseppe Esposito, Roberto Boni, Roberta Odorizzi, Daniela Caracciolo, Silvia Zamboni, Jacopo Mascherini, Barbara Catellani, Cristiano Guidetti, Stefano Di Sandro, Gian Piero Guerrini, Fabrizio Di Benedetto

**Affiliations:** 1 Hepato-Pancreato-Biliary Surgery and Liver Transplantation Unit, University Hospital of Modena “Policlinico”, University of Modena and Reggio Emilia, 41124 Modena, Italy; 2 Clinical and Experimental Medicine PhD Program, Department of Surgical, Medical and Dental Department of Morphological Sciences related to Transplant, Oncology and Regenerative Medicine, Modena, Italy

**Keywords:** HCC, MILS, Hepatectomy, Benign, Malignant, QoL

## Abstract

Advances in surgical techniques and perioperative care have improved outcomes in hepatic surgery, yet open liver resection (OLR) remains highly invasive, with substantial effects on recovery and quality of life (QoL). Robotic liver resection (RLR) offers a minimally invasive alternative with potential short-term benefits. While perioperative outcomes and early QoL have been studied, evidence on long-term health-related quality of life (HRQoL) is limited. This study evaluated long-term HRQoL after RLR versus OLR using the SF-36 questionnaire. A retrospective analysis was performed on patients undergoing robotic or open liver resection. HRQoL was assessed via SF-36 surveys. Clinical data included length of hospital stay (LOS), 90-day Comprehensive Complication Index (CCI), recurrence rates, and overall survival. Group comparisons used appropriate statistical tests. Of the eight SF-36 domains, three( vitality, general health, and mental health) were significantly improved in the RLR group. RLR patients had shorter LOS (5.5 ± 7.4 vs. 13.0 ± 11.2 days) and lower 90-day CCI (14.3 ± 17.2 vs. 26.7 ± 25.2). Bodily pain and physical functioning were similar between groups. RLR appears to provide long-term advantages in selected HRQoL domains, likely related to faster recovery, reduced morbidity, and enhanced body image. Oncologic outcomes were comparable, but RLR patients reported greater vitality, emotional stability, and social reintegration. In high-expertise centers, RLR may be a preferred option for benign and malignant liver disease.

## Introduction

Advancements in technology and modern medicine have significantly improved morbidity and mortality outcomes across all surgical specialties. Nevertheless, abdominal surgery, and particularly hepatic surgery performed via the classical open approach, can be highly invasive, leading to substantial impacts on patient performance status and long-term outcomes. Consequently, growing interest has emerged in the application of minimally invasive techniques for hepatic resections [1, 2]. In particular, robotic approach showed potential benefits both for the treatment of benign and malignant liver diseases [3, 4], with recent evidences in the field of living donor hepatectomy and liver transplantation, opening to new frontiers of the application of minimally invasive surgery in complex scenarios [5–10]. While the safety, oncological efficacy, and short-term benefits of robotic liver resection (RLR) have been acknowledged in the literature [11, 12], there is limited evidence regarding its long-term impact on health-related quality of life (HRQoL). The interest in studying quality of life (QoL) has increased in recent years, thanks to its ability to capture aspects of patient care and therapeutic goals previously not taken into account [13, 14]. QoL is subjective, multidimensional, and dynamic over time, and continuously fluctuates as it responds to life events. HRQoL can be defined as a multidimensional and subjective concept including at least four dimensions: physical dimension, functional level, emotional dimension, and social dimension. Furthermore, due to its subjectivity, to correctly define HRQoL we need to take into consideration the personal interpretation and expectancy that each patient gives to all dimensions.

This study aims to report HRQoL outcomes measured through the SF-36 Health Survey, administered via telephone interviews to patients that received either RLR or open liver resection (OLR) for benign or malignant diseases.

## Materials and methods

### Cohort selection

This observational study was based on retrospectively collected data from a prospectively maintained database. The cohort included patients who underwent liver resection between February 2014 and September 2022 at our Institution. Only patients who underwent either open or robotic liver resection were included. Robotic procedures were performed using the Da Vinci Surgical System (Si and Xi versions). No restrictions were applied regarding the indication for resection; thus, both malignant and benign pathologies were included in each group. Patients undergoing ALPPS (Associating Liver Partition and Portal vein ligation for Staged hepatectomy) were excluded from the analysis. The primary endpoints assessed were long-term health-related quality of life (HRQoL), evaluated using the SF-36 Health Survey, overall survival, recurrence rate (in malignant cases only), length of hospital stay, 30-day readmission rate, and 90-day Comprehensive Complication Index (CCI). Secondary perioperative variables included mean operative time, mean blood loss, and the number of major versus minor resections performed.

### The SF-36 survey

HRQoL was assessed using the validated Italian version of the 36-Item Short Form Health Survey (SF-36), originally developed by John E. Ware et al. in 1992 and translated/validated for the Italian population in 1998. The SF-36 consists of 36 items that evaluate eight dimensions of quality of life: (1) Limitations in physical activities due to health problems; (2) Limitations in social activities due to physical or emotional issues; (3) Limitations in usual role activities due to physical health problems; (4) Bodily pain; (5) General mental health (including psychological distress and well-being); (6) Limitations in usual role activities due to emotional problems; (7) Vitality (energy and fatigue); (8) General health perceptions. The survey is designed for self-administration by individuals aged ≥ 14 years or for administration by interviewers in person or via telephone—the latter was adopted in our study. Each answer was converted into a score ranging from 0 to 100. Scores for items within each dimension were averaged to yield a final subscale score (0–100), with higher scores indicating better health status. The SF-36 is widely employed in clinical research due to its brevity and strong psychometric properties and has proven effective in capturing QoL changes in patients undergoing abdominal surgery.

### Data collection

Telephone interviews were conducted between January 2023 and March 2024 to assess long-term postoperative QoL. Whenever possible, interviews were conducted directly with the patients. Patients who declined to participate or could not be reached were excluded. For each patient, additional clinical data—including surgical indication, postoperative complications, recurrence, and survival—were retrieved from our institutional electronic medical records. This included discharge summaries, radiological reports, and specialist outpatient clinic records. Short-term outcomes were assessed using the 90-day Comprehensive Complication Index (CCI), which aggregates all postoperative complications and weighs them according to severity based on the Clavien-Dindo classification. CCI scores were calculated using an online tool after recording all complications and treatments administered within 90 days of surgery. Where available, histological diagnoses were also collected and recorded.

### Statistical analysis

The Mann–Whitney U test was applied to compare continuous variables (e.g., CCI, age, follow-up duration, LOS, operative time, and intraoperative blood loss). Categorical variables (e.g., recurrence, resection type, survival status, readmission, and gender) were analyzed using the Chi-squared test.

SF-36 responses were scored according to the RAND methodology to obtain eight domain scores (0–100). Separate multivariable linear regression models were fitted for each RAND SF-36 domain. In the primary analysis, each domain score was modeled as the dependent variable, with surgical approach as the main independent variable and adjustment for age, sex and malignant indication, liver transplantation status, recurrence and 90-day Comprehensive Complication Index. Subgroup analyses were performed stratifying patients by disease type (benign vs. malignant).

## Results

### Population characteristics

During the study period, 303 liver resections were performed on 300 patients. Of these, 189 patients underwent robotic liver resection (RLR), and 111 underwent open liver resection (OLR). After excluding patients lost to follow-up (11 in the RLR group and 10 in the OLR group), those who died during follow-up (24 in the RLR group and 39 in the OLR group), and patients undergoing ALPPS (2 RLR, 5 OLR), a total of 226 patients were included in the final analysis, of whom 155 (70.8%) in the RLR group and 64 (29.2%) in the OLR group. Stratified by group, the mean age was 65.4 [IQR 55.1–72.1] in the RLR group and 62.0 [IQR 51.7–70.7]in the OLR group. Most of the cohort (141 patients, 64.3%) were male95 (61.3%)of whom in the RLR group and 46 (71.9%)in the OLR group. A total of 175 resections (79.9%) were performed for malignant lesions, 44 (20.1%) for benign lesions. Details regarding patient demographics and surgical indications are summarized in Table 1. The most frequent malignant diagnoses were Hepatocellular carcinoma (HCC) with 97 cases (82 RLR, 15 OLR), followed by Colorectal liver metastasis (CLM) with 31 cases (17 RLR, 14 OLR), whileintrahepatic cholangiocarcinoma (iCCA) accounted for 19 cases( 11 RLR, 8 OLR), 27 cases (14 RLR, 13 OLR) and Perihilar cholangiocarcinoma (pCCA) for 9 cases (6 RLR, 3 OLR). Combined, these accounted for 79.9% of all procedures. On the other hand, the three most common benign lesions were hepatic adenoma (13 cases, 12 RLR, 1 OLR) hepatic hemangioma (4 cases all RLR) and hydatid cysts (hepatic echinococcosis) with 6 cases (1 RLR, 5 OLR).

**Table 1:** Baseline characteristics of the study cohort

Characteristic	Overall (*N* = 219)	Open (*N* = 64)	Robotic (*N* = 155)	*p*-value
Age, years	65.0 [54.5–72.0]	62.0 [50.8–71.0]	65.4 [55.1–72.2]	0.200
Female sex, n (%)	78 (35.6)	18 (28.1)	60 (38.7)	0.200
Male sex, n (%)	141 (64.4)	46 (71.9)	95 (61.3)	
Charlson Comorbidity Index	20.9 [0.0–29.6]	20.9 [0.0–42.5]	0.0 [0.0–20.9]	**< 0.001**
Malignant disease, n (%)	175 (79.9)	50 (78.1)	125 (80.6)	0.700
cHCC-CCA, n (%)	1 (0.5)	0 (0.0)	1 (0.6)	
CRLM, n (%)	31 (14.2)	14 (21.9)	17 (11.0)	
HCC, n (%)	97 (44.3)	15 (23.4)	82 (52.9)	
iCCA, n (%)	19 (8.7)	8 (12.5)	11 (7.1)	
Other metastasis, n (%)	18 (8.2)	10 (15.6)	8 (5.2)	
pCCA, n (%)	9 (4.1)	3 (4.7)	6 (3.9)	
Benign disease, n (%)	44 (20.1)	14 (21.9)	30 (19.4)	
Adenoma, n (%)	13 (6.6)	1 (1.6)	12 (7.7)	
Hemangioma, n (%)	4 (2.0)	0 (0.0)	4 (2.6)	
Hydatid cyst, n (%)	6 (3.0)	5 (7.8)	1 (0.6)	

Data are presented as median [interquartile range] or number (%), as appropriate

### Surgical outcomes

 The overall mean follow-up duration was 51.7 months (± 30.1 months), and the average time between surgery and survey response was 47.3 months (± 27.8 months). Among patients who underwent resection for malignant disease, recurrence was observed in 51 cases (32,9%) within the 155 RLR subgroup. In the OLR group, 23 patients (35,9%) 38 experienced recurrence.

Liver transplantation (LT) performed after resection was more frequently observed in the RLR group compared to the OLR group (15.5% vs. 4.7%; *p* = 0.025) as part of a bridging strategy to LT.

Short-term perioperative outcomes were assessed using the 90-day Comprehensive Complication Index (CCI), hospital length of stay (LOS), and 30-day readmission rates. The median 90-day CCI value was 8.7 in the RLR group and 20.9 in the OLR group (*p*=0.08). For one patient in the OLR group, CCI could not be calculated. Median LOS was significantly shorter in the RLR group with 4 days )compared to 9days in the OLR group (*p* <0.001). At 30 days, the readmission rate was significantly lower in the RLR group (7.7%, 14 cases) compared to the OLR group (18.9%, 20 cases; *p* < 0.001). Comprehensive data on survival, CCI, LOS, and readmission rates are provided in Table 2.

**Table 2:** Outcomes

Variable	Open (*n* = 106)	Robotic (*n* = 183)	Total (*n* = 289)	*p*-value
Survival status				
Alive, n (%)	67 (63.2%)	159 (86.9%)	226 (78.2%)	
Deceased, n (%)	39 (36.8%)	24 (13.1%)	63 (21.8%)	
Perioperative outcomes				
90-day CCI, median (IQR)	20.9 [0–42.4]	8.7 [0–26.2]	12.5 [0–33.5]	0.08
Length of stay, days, median (IQR)	9 [6–15]	4 [3–7]	6 [3–10]	**< 0.001**
30-day readmission, n (%)	20 (18.9%)	14 (7.7%)	34 (11.8%)	**< 0.001**
Clinical outcomes				
Liver transplantation, n (%)	3 (4.7%)	24 (15.5%)	27 (12.3%)	0.025
Recurrence, n (%)	23 (35.9%)	51 (32.9%)	74 (33.8%)	0.8

Data are presented as median [interquartile range] or number (%), as appropriate

### Quality of Life (SF-36) assessment

All alive patients responded to the survey, translating into a response rate in the robotic group of 86.6%, compared to 63.4% of the open controls.

Health-related quality of life was assessed using the SF-36 questionnaire, with responses scored according to the RAND methodology.

In univariable analysis, the robotic group showed significantly higher scores in Vitality (75.0 [65.0–85.0] vs. 50.0 [35.0–65.0]; *p* < 0.001), Mental Health (84.0 [72.0–92.0] vs. 68.0 [52.0–84.0]; *p* < 0.001), and General Health (85.0 [70.0–95.0] vs. 70.0 [48.8–80.0]; *p* < 0.001). A statistically significant difference was also observed in Social Functioning (87.5 [62.5–100.0] vs. 100.0 [81.2–100.0]; *p* = 0.030) and Health Change (75.0 [50.0–100.0] vs. 50.0 [50.0–75.0]; *p* < 0.001).

No statistically significant differences were found in Physical Functioning, Role Physical, Role Emotional, or Bodily Pain (all *p* > 0.05).

In multivariable analysis, the robotic approach was independently associated with higher scores in General Health (β = + 13.26, 95% CI 6.62–19.89; *p* < 0.001), Vitality (β = + 20.46, 95% CI 14.93–25.99; *p* < 0.001), and Mental Health (β = + 12.34, 95% CI 6.99–17.68; *p* < 0.001), with no differences observed in the remaining domains (Table 3).

**Table 3:** SF-36 Quality of life outcomes

Abbrev.	Domain	OLR median [IQR]	RLR median [IQR]	Unadjusted *p*	Adjusted β (RLR vs. OLR)	95% CI	Adjusted *p*
PF	Physical Functioning	90.0 [75.0–100.0]	95.0 [55.0–100.0]	0.122	− 1.64	− 10.16 to 6.88	0.705
RP	Role Physical	75.0 [25.0–100.0]	100.0 [25.0–100.0]	0.275	1.14	− 10.39 to 12.66	0.846
BP	Bodily Pain	90.0 [67.5–100.0]	90.0 [70.0–100.0]	0.701	− 0.79	− 6.65 to 5.06	0.79
GH	General Health	70.0 [48.8–80.0]	85.0 [70.0–95.0]	< 0.001	13.26	6.62 to 19.89	**< 0.001**
VT	Vitality	50.0 [35.0–65.0]	75.0 [65.0–85.0]	< 0.001	20.46	14.93 to 25.99	**< 0.001**
SF	Social Functioning	100.0 [81.2–100.0]	87.5 [62.5–100.0]	0.03	− 5.67	− 13.01 to 1.67	0.129
RE	Role Emotional	100.0 [66.7–100.0]	100.0 [33.3–100.0]	0.436	− 7.42	− 18.46 to 3.63	0.187
MH	Mental Health	68.0 [52.0–84.0]	84.0 [72.0–92.0]	< 0.001	12.34	6.99 to 17.68	**< 0.001**

Data are presented as median [interquartile range]. *OLR* Open liver resection, *RLR* Robotic liver resection

To further explore the impact of surgical approach on quality of life, subgroup analyses were performed according to disease type.

In patients with benign disease, the robotic approach was associated with significantly higher scores in Role Physical (β = + 26.0, 95% CI 7.3–44.7; *p* < 0.01), General Health (β = + 20.7, 95% CI 8.7–32.7; *p* < 0.001), and Vitality (β = + 20.5, 95% CI 8.3–32.8; *p* < 0.001). No statistically significant differences were observed in Physical Functioning (β = + 9.2, 95% CI − 2.4 to 20.9; *p* > 0.05), Bodily Pain (β = − 4.4, 95% CI − 13.0 to 10.8; *p* > 0.05), Social Functioning (β = + 9.1, 95% CI − 0.8 to 19.0; *p* > 0.05), Role Emotional (β = + 7.4, 95% CI − 9.7 to 24.6; *p* > 0.05), or Mental Health (β = + 10.5, 95% CI − 0.7 to 21.8; *p* > 0.05).

In patients with malignant disease, the robotic approach was associated with significantly higher scores in General Health (β = + 14.2, 95% CI 6.4–21.9; *p* < 0.001), Vitality (β = + 22.7, 95% CI 16.7–28.7; *p* < 0.001), and Mental Health (β = + 15.4, 95% CI 9.4–21.3; *p* < 0.001). No statistically significant differences were observed in Physical Functioning (β = − 2.7, 95% CI − 12.7 to 7.3; *p* > 0.05), Role Physical (β = − 2.6, 95% CI − 14.0 to 10.7; *p* > 0.05), Bodily Pain (β = + 2.0, 95% CI − 4.7 to 8.7; *p* > 0.05), Social Functioning (β = − 6.0, 95% CI − 14.8 to 2.8; *p* > 0.05), and Role Emotional (β = − 6.0, 95% CI − 18.6 to 6.5; *p* > 0.05).

**Fig. 1:**
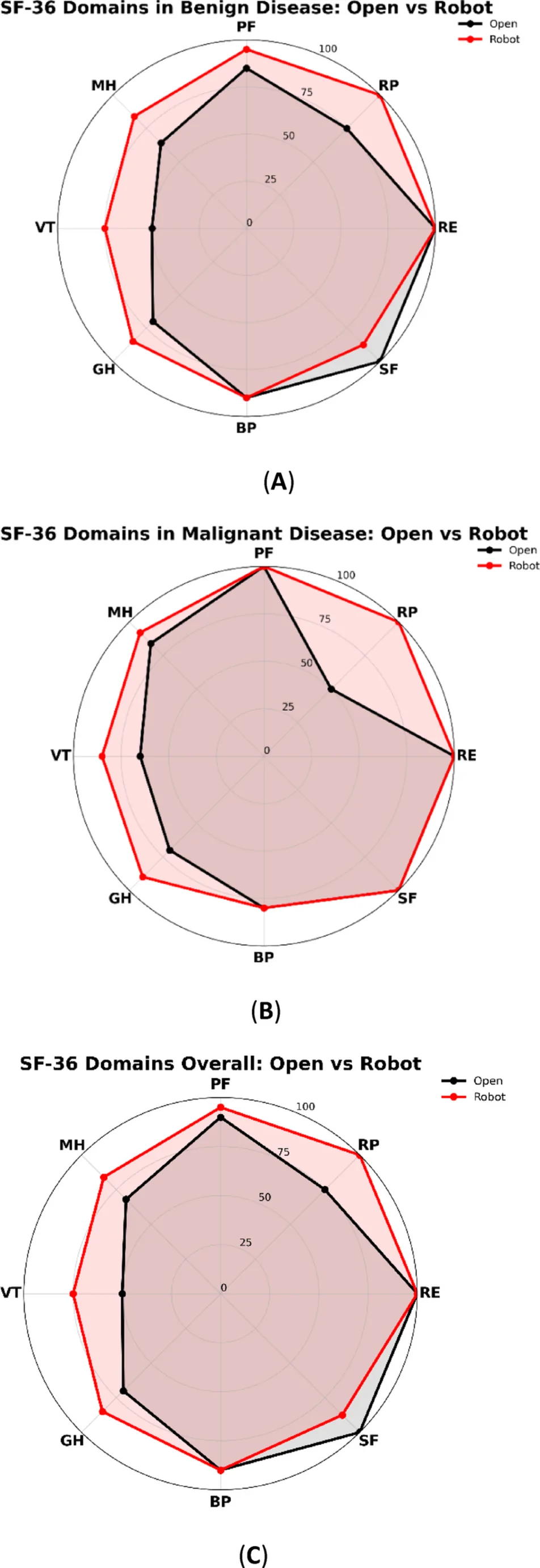
Radar graphics showing the distribution od SF-36 domains for patients in the robotic versus open approach groups for benign (**A**), malignant (**B**), and overall population (**C**)

The adjusted effect sizes for each domain, stratified by disease type, are illustrated in Fig. 1a-b-c.

## Discussion

HRQoL has emerged as a critical endpoint in the evaluation of modern surgical approaches, particularly in the context of minimally invasive liver surgery (MILS), where traditional outcomes such as morbidity and survival may not fully capture the patient experience.

In this study, HRQoL was assessed using the SF-36 questionnaire as a patient-reported outcome measure, allowing direct evaluation of patients’ perception of their postoperative health status [15]. The main finding is that, although several domains differed at univariable analysis, only vitality, mental health, and general health remained independently associated with the robotic approach after multivariable adjustment. No domain demonstrated superior outcomes in the open group.

These findings suggest that differences between surgical approaches are primarily observed in domains related to energy levels and psychological well-being, rather than in physical recovery. Indeed, no significant differences were observed in physical functioning, role physical, and bodily pain, indicating comparable long-term physical outcomes between approaches. Although social functioning differed in univariable analysis, this association was not confirmed after adjustment, suggesting that confounding factors may influence this domain.

Subgroup analyses provided further insight. In patients with benign disease, the robotic approach was associated with improvements in role physical, vitality, and general health, whereas in patients with malignant disease, differences were primarily observed in vitality, mental health, and general health. This pattern was consistent with the overall cohort and aligns with previous evidence indicating that underlying pathology plays a role in shaping postoperative quality of life [16].

The observation that only selected domains remained significant after adjustment highlights the importance of distinguishing between crude and independent associations. In contrast to prior studies, our analysis incorporates multivariable modeling, allowing a more refined assessment of the independent effect of surgical approach on HRQoL outcomes.

The results of the present study are consistent with those reported in the ORANGE II PLUS randomized trial, in which MILS was associated with improved patient-reported outcomes, particularly in functional and social domains, as well as reduced symptom burden [17]. Notably, in that trial, the largest differences in HRQoL were observed in the early postoperative period, with attenuation over time. In this context, the persistence of differences in selected domains such as vitality and mental health in the present study suggests that long-term effects of surgical approach may be subtle and domain-specific.

Importantly, SF-36 domains represent patient-reported outcomes, reflecting subjective perceptions of health rather than objective clinical measures. This distinction is particularly relevant, as the observed differences were confined to domains related to self-perception and psychological well-being, while traditional indicators of physical recovery were comparable between groups. These findings suggest that postoperative recovery may not be fully captured by conventional surgical outcomes alone.

We also explored the potential role of post-resection liver transplantation as a variable influencing long-term HRQoL. Although transplantation occurred more frequently in the open group, no significant association was observed between transplantation and SF-36 domain scores, suggesting that this variable did not confound the observed differences between surgical approaches.

The analysis of perioperative outcomes supports the established advantages of the robotic approach. While the difference in 90-day Comprehensive Complication Index did not reach statistical significance, the robotic group demonstrated a numerically lower complication burden, along with significantly shorter length of stay and lower 30-day readmission rates. These findings indicate differences in short-term recovery, although a direct causal relationship with long-term HRQoL cannot be established.

Although not directly measured in this study, the minimally invasive nature of the robotic approach may influence patients’ perception of recovery. Factors such as reduced surgical trauma and limited visible impact may contribute to differences in patient-reported domains, particularly those related to vitality and mental health. However, this interpretation remains speculative.

This study has several limitations. Its retrospective single-center design may limit the generalizability of the findings, and the observational nature introduces potential selection bias. Quality-of-life assessment was limited to patients alive at follow-up, introducing an inherent survivorship bias, as patients with poorer outcomes were not represented. Furthermore, the absence of preoperative baseline HRQoL data precludes assessment of longitudinal changes. The use of telephone interviews may also introduce reporting bias. Finally, the use of a generic QoL instrument may limit sensitivity in detecting disease-specific differences, in particular cancer-specific implications. Nonetheless, this study expands the current body of literature on a sensitive topic, contributing to a deeper interpretation of the impact of MILS.While these findings are promising, further prospective and multicentric studies with baseline and longitudinal HRQoL assessment are necessary to validate the generalizability and causality of these associations.

## Conclusion

This study reinforces the safety, feasibility, and perioperative advantages of robotic liver resection for both benign and malignant liver lesions, in line with existing literature. More importantly, it suggests a potential long-term benefit in patient-reported HRQoL, particularly in domains such as vitality, emotional well-being, social functioning, and general health.

## Data Availability

Raw data are available upon reasonable request to the corresponding author.
